# The Evaluation of Different Polishing Techniques' Effects on the Post-operative External Staining of Enamel in Primary and Permanent Tooth

**DOI:** 10.7759/cureus.39690

**Published:** 2023-05-30

**Authors:** Ömer Faruk Okumuş, Recep Orbak, Yerda Özkan Karasu, Pınar Gül

**Affiliations:** 1 Department of Periodontology, Erzincan University Faculty of Dentistry, Erzincan, TUR; 2 Department of Periodontology, Atatürk University Faculty of Dentistry, Erzurum, TUR; 3 Department of Restorative Dentistry, Atatürk University Faculty of Dentistry, Erzurum, TUR

**Keywords:** post-op discoloration, enamel discoloration, occupational regulation, polishing brush, air polishing, rubber cup, dental polishing, tooth discoloration

## Abstract

Objective

This study aimed to examine patient complaints on recoloration development after polishing applications in primary and permanent teeth that differed in enamel composition and to determine the ideal polishing method.

Methods

A total of 30 permanent upper incisors and 30 primary molars were randomly divided into three groups of 10 using three different polishing techniques. Each polishing method (rubber, brush, and air polishing) was applied to the test surface of its own group. Milk and coffee were used in the coloring processes. A spectrophotometer was used for color measurements. Color change (∆E) was calculated between control and test surfaces and between the three measurement points.

Results

In the primary teeth’s test surfaces, the rubber and brush groups were significantly more colored than the air-polishing group, when compared between after polishing and after coloration (p ˂ 0.05). Furthermore, the color difference of the permanent teeth between the initial measurements and after coloration was significantly higher in the rubber group’s test surface compared to the air-polished group (p ˂ 0.05). The average ∆E values in both primary and permanent teeth were as follows: rubber > brush > air polishing.

Conclusions

Compared to rubber or brush polishing, air polishing seems safer to avoid predisposition to postoperative enamel discoloration. Primary teeth are more colored than permanent teeth. The effect of polishing on postoperative coloring should always be considered, and air polishing should be preferred whenever possible.

## Introduction

Polishing is often applied after dental scaling to remove tooth discoloration and debris. The discolorations that can be removed by polishing are of the external tooth coloration type and occur on the outer surface [1].

External tooth discoloration occurs when chromogenic substances are deposited on the pellicle, plaque, or calcified attachments on the tooth surface [2]. Attachments originating from the precipitation of glycoproteins on the tooth surface form the basis for this type of discoloration [2].

External coloration, which can be in various colors such as brown, yellow, and black, creates aesthetic discomfort. However, this type of discoloration does not have a pathological aspect in terms of health, and the purpose of its removal is entirely cosmetic.

There are different polishing techniques preferred in today’s dental clinics. The most frequently used ones are polishing with rubber or a brush. These techniques are performed by applying the polishing rubber or brush to the tooth surface using an abrasive polishing paste with the help of a rotary instrument. Factors such as the abrasiveness of the polishing paste used, the rotation speed of the rotary instrument, the application pressure, and the application time are crucial for removing the discoloration and avoiding undesirable situations such as increased temperature and roughness on the surface [3].

On the other hand, air polishing is a newer technique than rubber or brush polishing, and it has become widespread in clinical use in recent years. Air polishing is a stain removal method performed with a device that sprays abrasive powder and water, such as sodium bicarbonate, glycine, or calcium carbonate, at a certain pressure [4]. Factors such as the distance from the tip of the device to the tooth, the application angle, and the spray pressure are decisive in stain removal, affecting the tooth and surrounding tissues [4].

Polishing has some harmful effects on dental tissues, restorations, and gingiva. For this reason, the balance between benefit and harm should be considered before the procedure. In addition, contraindications for polishing with rubber and brush are defined in the literature [5]. One of these contraindications is the absence of any external coloration.

In case of inflammation in the gingiva, polishing causes further tissue destruction because the particles in the polishing paste invade the gingiva and irritate the tissue [6]. The superficial layer of fluoride-rich enamel is removed when polished with rubber [5]. This layer is removed particularly in patients with a high risk for caries and may have more negative results [7]. Compared to the tooth’s enamel, dentin and cement show more wear due to polishing. For this reason, polishing should be avoided, especially on root surfaces; otherwise, tooth sensitivity may develop [5].

Pulp damage may develop due to heat generation by inappropriate polishing made with rubber and brush, which is hazardous, especially for primary teeth with large pulps [5]. Furthermore, polishing should be avoided as much as possible since the wear amount will be higher in newly erupted and primary teeth that are not sufficiently mineralized [5].

In air polishing, the risk of material loss is higher on dentin and cementum surfaces rather than enamel tissue [8], causing changes including an increase in surface roughness and discoloration, and the appearance of marginal adaptation disorder on restoration surfaces [9,10].

The effects of these frequently applied polishing methods on tooth surfaces have been an area of interest for many researchers. Studies show frequent use of profilometric devices for surface roughness measurements and scanning electron microscope (SEM) imaging devices for surface topography [11-13]. These studies have clarified the abrasive effect of polishing on dental tissues. Except for the abrasive effects of polishing on dental tissues, no study shows an increased tendency to discoloration due to polishing on the tooth surface.

Feedback shows that some patients had faster and more intense coloration development after the polishing applications, which led to the need to question existing polishing processes. Therefore, this study aimed to examine patient complaints on recoloration development after polishing applications in primary and permanent teeth that differed in enamel composition and to determine the ideal polishing method. Additionally, we sought to evaluate the effect of using milk on coloration due to its glycoprotein content in the in vitro coloring processes.

## Materials and methods

This study was carried out with decision number 12 of the Atatürk University Faculty of Dentistry Research Ethics Committee meeting dated January 15, 2019.

The teeth used in the study were collected with patients’ consent.

A total of 30 permanent upper incisors and 30 primary molars were used in the study. Exclusion criteria were caries affecting the enamel layer, developmental or acquired deformations, and restorations. Primary and permanent teeth were randomly divided into three groups of 10, using three different polishing techniques. Randomization was done by two different researchers who did the experiment. The polishing method of each group was determined after the samples were separated into 10 pieces. One of the researchers was unaware of the polishing method that would be assigned to the groups she formed while randomly handing out the samples. The other researcher randomly assigned three different polishing methods to the groups created.

In an additional study, in which we evaluated the contribution of milk to coloration during coloring processes, 20 upper permanent incisor samples were randomly divided into two groups of 10, independent of the above six groups.

Preparation of samples

The teeth's roots were removed by cutting 2 mm apical to the cementoenamel junction. The crowns of the obtained teeth were fixed in silicone molds by trying to position them parallel to the ground plane, leaving the vestibular surfaces exposed. All vestibular surfaces of the samples were cleaned of debris on the surface using rubber and paste for five seconds to ensure standardization. The samples’ vestibular surfaces

were handled as two separate plains, right and left; the right side was determined as the test surface and the left side as the control surface.

Polishing procedure

The control surfaces were sealed with celluloid tape and isolated, while the samples’ test surfaces were polished. In the permanent and primary tooth groups polished with rubber, the rubber (REF: EPY003A, Stoddard®, Letchworth, United Kingdom) and polishing paste (Detartrine, Septodont®, Saint-Maur, France) were used for five seconds at 2500 rpm.

In the permanent and primary tooth groups polished with a brush, the brush (RubyBrush, İnci Dental®, İstanbul, Turkey) with polishing paste (Detartrine, Septodont®, Saint-Maur, France) was applied for five seconds at 2500 rpm.

Air polishing was applied with an air-polishing device (MyFlow, DÜRR Dental AG., Germany) and air-polishing powder (Lunos Prophylaxe Pulver DÜRR Dental AG., Germany) to permanent and primary tooth groups. The application lasted for five seconds by adjusting the tip of the air polisher at a 45° angle with 5 mm from the surface.

After polishing, the remaining paste and air-polishing powder residues on the teeth were removed by washing them with physiological saline.

Color measurement

A spectrophotometer (Spectro Shade TM MICRO, MHT Optic Research AG, Milan, Italy) was used for color measurements. The device was calibrated as recommended by the company before analysis. The colorimeter researcher was unaware of the type of polish applied to the samples. Color measurements were made under daylight on a standard white background. Data were recorded according to the CIELAB, (L*a*b*) L (black-white), a (red-green), and b (blue-yellow).

Initial color measurements were performed first. This process was done after the samples were prepared and before applying selected polishing. Color measurements were made separately from each sample’s test and control surfaces and recorded.

The second color measurements were carried out for each test surface after the determined polishing process was complete. Since there would be no change in the control surface, this measurement was made only on the polished test surfaces.

The third color measurement was performed after applying the coloring procedure to the samples. The color data of each colored sample were taken separately from test and control surfaces and recorded.

Coloring procedure

Coloring was applied after polishing. The following staining procedure was repeated every day for seven days to establish the external coloration. First, 1.5 L of fresh cow’s milk was added to the partitioned test cup to form a pellicle layer on the sample surfaces, fully covering the samples. The samples were kept in milk for two hours. After the milk was completely removed from the test cup, a standard Turkish coffee solution (150 g of Turkish coffee per 1500 mL of water) was prepared. The prepared coffee solution was added to the test cup to cover the surface of the samples (1.5 L). Samples were moved out of the partitioned test cup during solution changes. In order not to damage the pellicle layer that will form on the surface of the samples, no rinsing process was applied to the samples. Only the partitioned test cup was washed and dried during solution changes. This procedure was repeated every 24 hours and applied for seven days.

Experiment protocol for the effect of milk on coloration

This independent study investigated the effect of milk pellicle on external coloration. An additional 20 samples were prepared independently of the main experiment. Of the two groups, each consisting of 10 teeth, the samples in the experimental group were subjected to the coloring procedures using milk as described above. On the other hand, the teeth in the control group were kept in distilled water instead of milk. Color measurements were taken from the experimental and control groups before and after the coloring procedures.

Calculation of color change (∆E)

The ∆E values expressing the color change between the two surfaces were calculated using the below formula:

∆L = L2* − L1* (ΔL = brightness values)

∆a = a2* − a1* (Δa = determines the difference in the red-green scale)

∆b = b2* − b1* (Δb = determines the difference in the blue-yellow scale)

∆E = [(∆L)2 + (∆a)2 + (∆b)2]^½^ (ΔE = color difference).

The color change parameter ∆E was considered in the statistical evaluation and comparisons between the groups. The ∆E color change value was calculated using the data of the two surfaces at the three measurement points.

∆E Values Between Test and Control Surfaces

**Figure 1:**
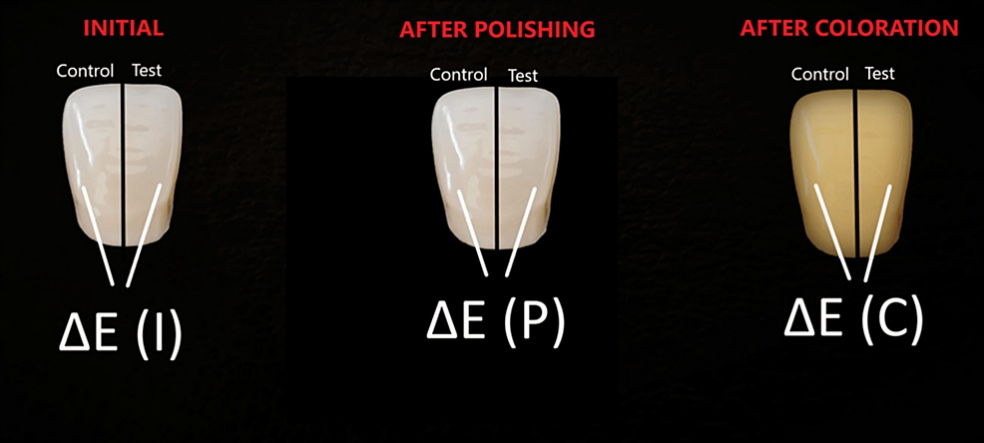
∆E values between test and control groups ∆E (I), color difference between the test and control surface at the initial stage; ∆E (P), color difference between the test and control surface after polishing; ∆E (C), color difference between the test and control surface after coloring

First, the ∆E color difference values in test and control surfaces of each sample were calculated. The ∆E values were calculated separately for each sample in all three time points. Thus, a total of three ∆E values were obtained for each sample, one at each time point (Figure 1).

∆E Values Between the Three Measurement Points

**Figure 2:**
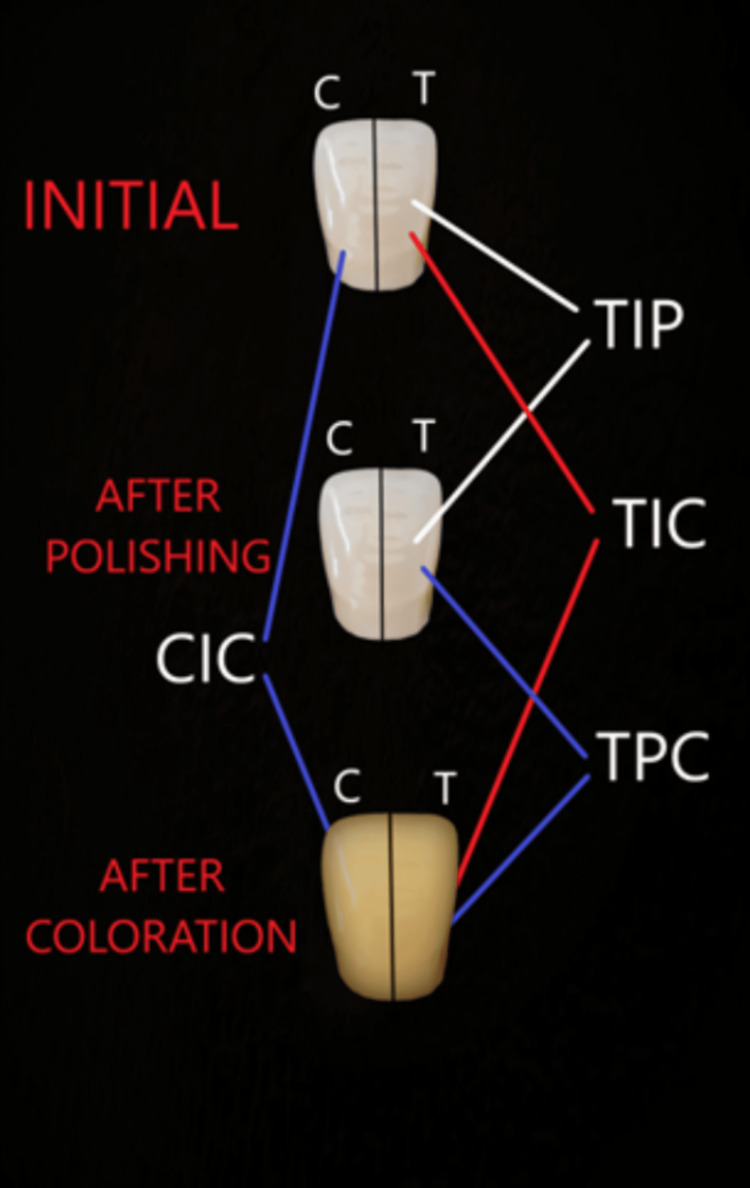
∆E values between the measurement times TIP, color change of the test surface compared between initial and after polishing; TIC, color change of the test surface compared between initial and after coloration; TPC, color change of the test surface compared between after polishing and after coloring; CIC, color change of the test surface compared between initial and after coloration

In addition, the samples’ test and control surfaces were handled independently of each other. The color change (∆E) of the same surface between different time points was calculated (Figure 2).

Statistical analysis

Statistical analysis was performed using the IBM SPSS Statistics, version 18.0 (IBM Corp., Chicago, IL, USA) package program. The Kolmogorov-Smirnov test was used to determine the data distribution. The Friedman test was used to compare the ∆E values of test and control surfaces after initial polishing and coloring and those of the test surfaces at each time point. In addition, the Wilcoxon signed-ranks test was used to determine the source in case of any differences. The Kruskal-Wallis test was used to compare the polishing systems in permanent and primary teeth at each time point, and the Mann-Whitney U test was used to determine the source in case of differences (α = 0.05).

## Results

Comparisons between test and control surfaces

Comparisons between test and control surfaces are shown in Table 1.

In primary teeth polished with rubber, a significant ∆E increase was observed between test and control surfaces after coloration compared to the baseline values (p ˂ 0.05). However, in all other groups, ∆E increases were insignificant after coloration between test and control surfaces compared to baseline values (p ˃ 0.05) (Table 1).

**Table 1:** Comparison of ∆E values between the control and test surfaces in different groups The difference in superscript lowercase letters indicates statistical significance (p < 0.05) within the row, while the difference in uppercase letters shows significance within the column R, rubber polishing; B, brush polishing; A, air polishing

	Polishing	Initial	After polishing	After coloration
Permanent teeth	R	2.86±2.98^aA^	1.46±0.62^aA^	2.59±1.30^aA^
	B	2.21±3.26^aA^	1.46±0.73^aA^	2.87±1.60^aA^
	A	1.45±1.08^aA^	1.66±1.48^aA^	2.54±2.17^aA^
Primary teeth	R	2.21±1.49^aA^	1.52±0.58^aA^	3.54±2.10^bA^
	B	2.15±0.80^aA^	2.22±1.33^aA^	3.81±2.47^aA^
	A	2.27±1.46^aA^	2.20±1.35^aA^	5.32±4.45^aA^

Measurements between time points

Comparisons between the time points are shown in Table 2.

In primary teeth, when compared between the initial measurements and after coloring, the rubber and brush groups’ test surfaces were significantly more colored than the air-polishing group’s test surfaces (p ˂ 0.05). When compared between the initial measurements and after coloring, the test surface of the rubber-polished permanent teeth was significantly more colored than the air-polished test surface (p ˂ 0.05). There were no significant color differences between the initial measurements and after coloration in the control surfaces of all groups (p > 0.05).

**Table 2:** Comparison of the ∆E values between the measurement points in different groups The difference in superscript lowercase letters indicates statistical significance (p < 0.05) within the row, while the difference in uppercase letters shows significance (p < 0.05) within the column R, rubber polishing; B, brush polishing; A, air polishing; TIP, color change of the test surface compared between initial and after polishing; TIC, color change of the test surface compared between initial and after coloration; TPC; color change of the test surface compared between after polishing and after coloring; CIC, color change of the test surface compared between initial and after coloration

	Polishing	TIP	TPC	TIC	CIC
Permanent teeth	R	2.71±2.42^aA^	22.74±4.03^b^^ABC^	22.12±3.59^bAC ^	21.63±5.48^bA^
	B	4.34±5.76^aA^	20.07±5.07^b^^ABC^	17.72±5.30^cAB^	18.08±5.71^bcA^
	A	2.23±1.03^aA^	18.47±5.08^b^^AC^	16.86±4.73^cB^	17.16±5.00^bcA^
Primary teeth	R	1.76±1.52^aA^	23.99±5.21^bB ^	22.97±5.32^cC ^	21.45±4.60^cA^
	B	2.18±0.95^aA^	22.67±5.06^bc^^AB ^	21.37±5.00^cABC^	23.00±5.92^cA^
	A	1.20±1.08^aA^	19.14±7.49^b^^C ^	19.11±6.83^cB ^	18.16±6.54^bcA^

The effect of milk

The mean ∆E value of the milk-coloring group was significantly higher than the control group (p ˂ 0.001).

## Discussion

This study evaluated the contribution of three different polishing methods applied in clinical practice to external discoloration on tooth enamel. Based on the ∆E color change values between test and control surfaces and between time points, significant results were obtained for the development of coloration. However, the rubber polishing difference between test and control surfaces was significant only in primary teeth.

Although no study shows that polishing techniques cause discoloration in tooth tissues, these techniques are thought to create roughness on the enamel surface [5,14]. In some studies, a connection was established between surface roughness and color change [15,16].

There are studies showing a significant relationship between the roughness of the enamel surface and the CIELAB color system that we used in our study [16,17]. We evaluated our findings on the effect of polishing on enamel tissue discoloration based on the significant correlation of roughness with color change, which was proven in previous studies.

When the analysis based on the ∆E values formed between test and control surfaces was examined, a significant increase was found in the rubber-polished group in the primary teeth after the coloring procedures.

In a study by Camboni and Donnet, the effects of air and rubber polishing using different polishing pastes on enamel were compared with SEM imaging [12]. Accordingly, while there was no difference in air-polished roughness between test and control surfaces, the test surfaces were roughened more than the control surfaces in rubber polishing [12]. Another study conducted in 1987 concluded that air polishing has a much less abrasive effect on enamel than rubber and pumice powder polishing [8].

If we evaluate the results between the time points, our study’s test surface of the rubber-polished group on permanent teeth was significantly more colored than the air-polished test surface. Similar results were obtained in the same evaluation that we made for primary teeth. The fact that rubber-polished surfaces are more colored than air-polished surfaces in both permanent and primary teeth is consistent with the results of previous studies. On the other hand, another study showed no difference between the two polishing techniques in terms of surface roughness [11].

In our study, the primary teeth’s test surface of the brush-polished group was significantly more colored after the polishing than the air-polishing group. In a study where Hosoya and Johnston compared both polishing methods in extracted primary teeth, while air polishing did not develop enamel roughness, roughness developed on brush and pumice-applied surfaces [13]. We think that brush polishing increases the coloration potential by causing more changes in the enamel surface than air polishing.

The numerical interpretation of the ∆E values yields significant findings. Increases and decreases in values allow objective interpretation of subjective findings in colorations. There are studies showing that the color change (∆E) perceived by the human eye is equal to 1 [18,19]. Some studies show that this detection limit can differ between 2 and 3 [20,21]. Additionally, there are studies suggesting that the ∆E value (which is clinically intolerable and requires intervention) is equal to or greater than 2.75 or 3.7 [19,22].

Although they reached no statistical significance, the mean after-polish coloring ∆E values were higher on the surfaces polished with rubber than with a brush. This excess is above the perceptible limit. In addition, according to the same evaluation, the average ∆E values are much higher in the rubber-polished group than in the air-polished group, and this excess is at a level that requires clinical intervention.

If the data we have obtained are sorted by average ∆E values in both primary and permanent teeth concerning postoperative coloring, then they are as follows: rubber > brush > air polishing. Thus, the highest values in both tooth groups were obtained in the rubber-polished groups.

In addition, considering these numerical values, all three polishing methods gave higher scores in primary teeth compared to permanent teeth. However, these values, which were higher in primary teeth, were insignificant for all three groups (p > 0.05). Our study obtaining higher mean ∆E values in primary teeth can be explained by the mineral content of the primary teeth’s enamel being lower than that of the permanent teeth and that it is less resistant to abrasion. In addition, there are studies to support the idea that polishing should not be applied to primary teeth unless it is mandatory [5,7].

There was no statistical difference between the color changes occurring after the coloring process on the control surfaces of all groups compared to the baseline (p > 0.05).

This finding supports the reliability of our study. While significant differences could be detected between some of the test surfaces that we polished, the inability to detect any difference between the untreated control surfaces proves that the applied polishing processes can affect the coloration potential by causing some changes on the enamel surface.

In previous external coloring experiments, no study used milk to create a pellicle layer on the tooth surface. However, some studies can indirectly explain the contribution of milk to the external discoloration mechanism on the tooth surface.

Some in vitro studies show that milk proteins, like salivary proteins, form a pellicle layer on the hydroxyapatite surface of the enamel [23,24]. These studies showed that the pellicle layer required to create coloring be formed on the tooth with milk; however, this pellicle layer must be able to bind the coloring agents in tea and coffee, just like the proteins of the salivary pellicle. In 1963, Brown and Wright determined that the coloring agents in tea and coffee bind with the proteins in the milk content [25].

In our study, we evaluated the effectiveness of milk in external coloring processes with the formed pellicle layer. More coloration occurring with milk is compatible with the results of previous studies. It can be inferred that milk can be used in external coloring experiments.

The main limitation of this study is that it is an in vitro study. Randomized controlled clinical studies with larger sample sizes are needed to evaluate the discoloration of dental tissues after polishing. In addition, the effectiveness of using milk to obtain a pellicle layer in external coloring processes should be supported by future studies.

## Conclusions

Our results show that polishing the enamel surface may cause changes in the surface’s coloration potential. Therefore, except for clinically mandatory reasons, it seems important not to apply polishing to avoid its traumatic effects. If polishing is required, air polishing should be preferred whenever possible. In addition, it should be noted that primary teeth may be more affected by the harmful effects of polishing techniques than permanent teeth.
